# A ketogenic‐promoting beverage acutely elevates cardiac function and myocardial blood flow compared to placebo in adults: A cardiac MRI investigation

**DOI:** 10.14814/phy2.70208

**Published:** 2025-03-18

**Authors:** Christopher D. Crabtree, Yue Pan, Preethi Chandrasekaran, Yuwei Wang, Debbie Scandling, Teryn Bedell, Justen Stoner, Drew Decker, Madison Kackley, Bradley Robinson, Alex Buga, Yuchi Han, Jeff Volek, Orlando P. Simonetti

**Affiliations:** ^1^ Department of Radiology The Ohio State University Columbus Ohio USA; ^2^ Department of Human Sciences The Ohio State University Columbus Ohio USA; ^3^ Department of Biomedical Engineering The Ohio State University Columbus Ohio USA; ^4^ Division of Cardiovascular Medicine, Department of Internal Medicine The Ohio State University Columbus Ohio USA

**Keywords:** cardiac function, cardiac MRI, ketones, metabolic imaging, myocardial perfusion

## Abstract

Increasing evidence suggests cardiac function improves in healthy and failing hearts alongside circulating ketones (1–4 mM). This study characterized cardiac function and blood flow responses to a ketogenic beverage compared to a volume/calorie matched placebo with repeated imaging over 120 min. This was a two‐group, placebo‐controlled, acute cardiac imaging study. Adults without cardiac abnormalities underwent baseline cardiac MRI including quantitative myocardial perfusion to measure myocardial blood flow (MBF). Subjects consumed 50 g of a ketogenic‐promoting beverage [bis‐hexanoyl R‐1‐3‐butanediol (BH‐BD)] (BH‐BD; *n* = 11) or a calorically/volume‐matched lipid‐based placebo (PL; *n* = 10) with cardiac MRI every 15–30 min. Following 120 min, subjects underwent a final scan including MBF measurement. R‐BHB and glucose were measured at every timepoint. 120 min following BH‐BD consumption, R‐BHB reached 2.1 mM. Cardiac output (CO) was elevated compared to PL (*p <* 0.05) and increased +31% 120 min after BH‐BD ingestion (*p <* 0.001). CO elevation was due to increased stroke volume (+11%; *p* = 0.02) and heart rate (+22%; *p <* 0.001). MBF increased 29% from baseline (*p* < 0.001). PL did not induce differences in cardiac parameters. 50 g BH‐BD ingestion achieves exogenous ketosis and is associated with elevated MBF and CO providing evidence supporting their use as a therapeutic clinical agent.

## 1 INTRODUCTION

In a healthy individual consuming a traditional western mixed diet, 60%–90% of the energy requirements of the heart are met by fatty acid oxidation. However, individual cardiomyocytes are metabolically flexible and rapidly adapt to changing substrate availability (Stanley et al., 2005). This includes utilization of ketones (Vanitallie & Nufert, 2003) as fuel (Luong et al., 2023) proportional to their circulating concentrations (Bassenge et al., 1965; Murashige et al., 2020). The failing heart is less metabolically flexible, characterized by impairment of fatty acid oxidation (Azevedo et al., 2013; Sochor et al., 1986; Yurista et al., 2022) with an increase in myocardial ketone uptake and use (Aubert et al., 2016; Bedi et al., 2016); it remains unclear if this is adaptive or maladaptive. Regardless of the functional status of the heart, the availability of ketones appears to confer metabolic and functional advantages to the myocardium (Ferrannini, Mark, & Mayoux, 2016; Sato et al., 1995; Veech, 2004).

Achieving beneficial circulating ketone concentration has become a therapeutic target of investigation for those suffering from various chronic diseases, including cardiovascular disease (Monzo et al., 2021; O'Brien & Tian, 2021; Takahara et al., 2022; Yurista et al., 2021). Much of the recent interest in ketones stems from sodium‐glucose cotransporter 2 inhibitor (SGLT2i) trials demonstrating mild ketosis is associated with improved outcomes in people with CVD, including a 40% reduction in cardiovascular mortality in people with type 2 diabetes (Ferrannini, Mark, & Mayoux, 2016; Taegtmeyer, 2016). These improvements were concurrent with elevated plasma R‐beta‐hydroxybutyrate concentration [R‐BHB] of approximately 0.5 mM (Ferrannini, Baldi, et al., 2016; Polidori et al., 2018), providing evidence for a possible therapeutic benefit of nutritional ketosis (Ferrannini, Mark, & Mayoux, 2016).

In multiple human studies, intravenous infusion of a ketone salt (sodium BHB) compared to placebo led to significant increases in cardiac output (CO), stroke volume (SV), heart rate (HR), and left ventricular (LV) ejection fraction (EF) following two (Nielsen et al., 2023), three (Nielsen et al., 2019), and three and a half hours (Gormsen et al., 2017) of infusion. Additionally, BHB infusion elicited a dose–response effect on CO as measured by echocardiography in people with heart failure (HF) and their healthy age‐matched volunteers (Nielsen et al., 2019). Myocardial blood flow (MBF) measured by ^11^C‐palmitate positron emission tomography (PET) also increased following ketone infusion in both people with HF and healthy volunteers (Gormsen et al., 2017; Nielsen et al., 2019).

While infusion studies have demonstrated the acute cardiovascular effects of elevated blood ketones, a more practical noninvasive method of achieving exogenous ketosis involves ingestion of exogenous ketones or pro‐ketones that are converted to ketones through classical and nonclassical hepatic pathways (Crabtree et al., 2023). Changes in LV function in healthy younger adults, similar to those observed in the infusion studies, have been reported up to 1 h post‐ingestion of exogenous ketone monoesters as measured by echocardiography (Selvaraj et al., 2022) and by cardiovascular magnetic resonance (cardiac MRI) compared to a water placebo (Oneglia et al., 2023). Given that ketones are theorized to benefit a multitude of cardiac disease populations (Takahara et al., 2022) including both left and right heart disease as well as ischemia (Makievskaya et al., 2023), there is a need to evaluate biventricular function as well as myocardial blood flow following ingestion of exogeneous ketones.

Based on kinetic testing, we chose to study an exogenous ketone diester formulation consisting of bis‐hexanoyl R‐1‐3‐butanediol (BH‐BD) that elevates R‐BHB for a period of 3 or more hours while reaching blood concentrations up to 3 mM (Selvaraj et al., 2022), depending on serving size (Crabtree et al., 2023). We hypothesized that elevated R‐BHB following BH‐BD ingestion would be associated with increased biventricular function, LV contractile strain, and myocardial blood flow (MBF); advanced cardiac MRI techniques can be used to measure the time course of these parameters to gain further insight into the temporal cardiovascular response. In this study, we sought to simultaneously investigate the effect of acute ketone diester supplementation on changes in CO and quantitative MBF using cardiac MRI over the course of 2 h compared to an energy and volume‐matched placebo to adequately control for postprandial effects on cardiac function (Hauser et al., 2016; Oneglia et al., 2023).

## 2 METHODS

### 2.1 Study design

**FIGURE 1:**
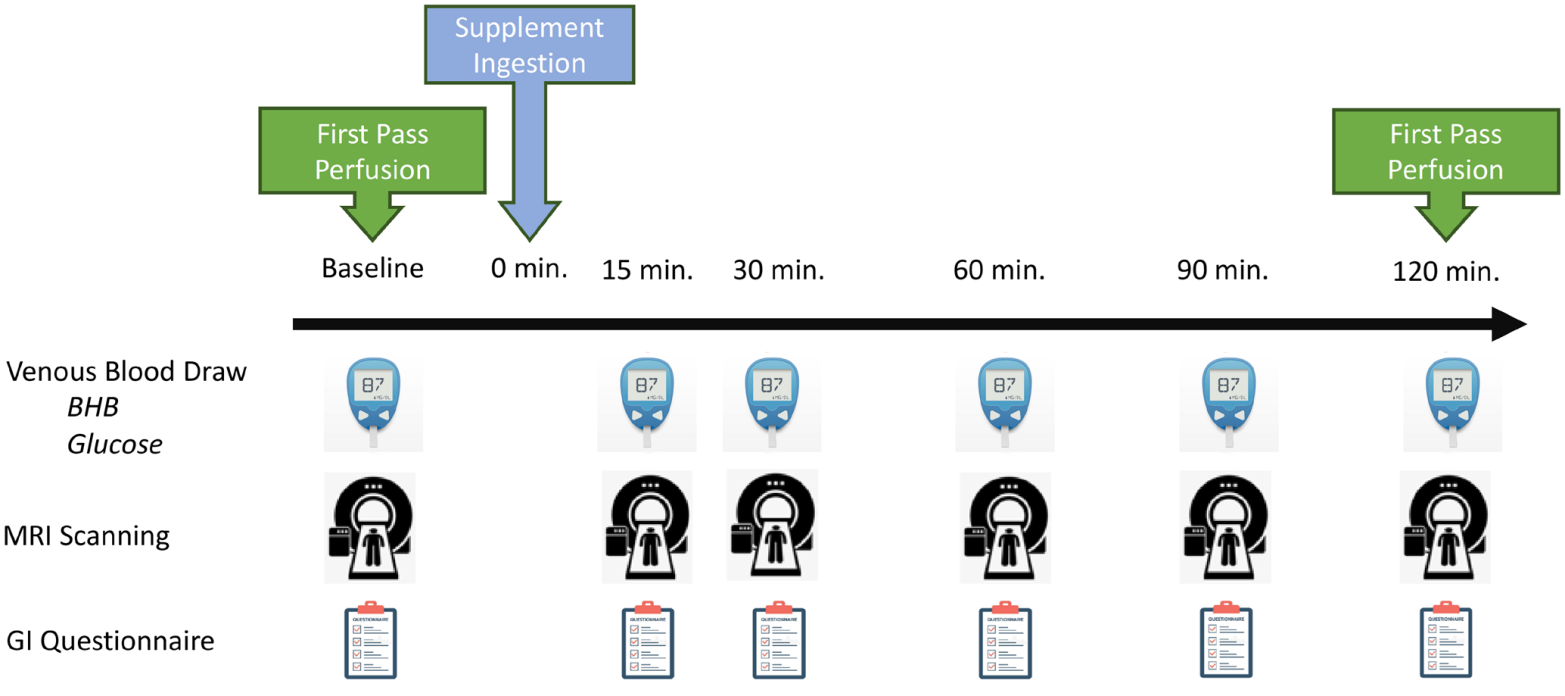
Study timeline. Subjects arrived at the imaging facility following an overnight fast and underwent baseline imaging. Following baseline, subjects consumed the active or placebo beverage, and underwent repeated cardiac imaging and venous blood draws every 15–30 min.

This study was approved by the Institutional Review Board at the Ohio State University and all participants provided written informed consent prior to participation (2021H0425). Between March 2022 and June 2023, we enrolled participants with normal cardiac systolic function and without known cardiovascular disease aged 18–65 years old with a BMI between 18 and 30 kg/m^2^ and a bodyweight >68 kg. We excluded potential subjects with contraindications to MRI or gadolinium contrast agent (claustrophobic, pregnant, breastfeeding, implanted devices, or glomerular filtration rate (GFR) <60 mL/min/1.73 m^2^), allergies to components in either test product, history of alcoholism, currently smoking, or taking prescription medications or over the counter medications other than those taken as nutritional supplements. We also excluded subjects currently consuming a ketogenic diet (daily carbohydrate intake ≤50 g/day) or ketogenic supplements, to avoid any potential changes in response caused by elevated baseline circulating ketones. Our initial goal was to recruit a single group of 10 participants and study the cardiac response to acute BH‐BD ingestion. After completing the majority of those participants and determining a robust improvement in cardiac function, we decided it would be important to control for effects of fluid and/or nutrient ingestion. Thus, we amended our protocol to recruit a separate matched cohort to undergo the exact same testing but ingest a caloric‐ and fluid‐matched placebo. In total, 21 subjects underwent an acute, single visit study comparing a BH‐BD (*n* = 11) to a placebo (*n* = 10) beverage with repeated cardiac MRI imaging and blood collection during a 2‐h period post ingestion (Figure 1). The placebo was a calorie and fluid volume‐matched fat‐based beverage used to control for postprandial effects on cardiovascular function (Hauser et al., 2016; Oneglia et al., 2023).

### 2.2 Study beverages

This study featured two test beverages: a placebo and a ketogenic promoting beverage. The ketone beverage contained 25 g of active ingredient BH‐BD/serving in a chocolate‐flavored matrix (whey, whey protein concentrate, modified gum acacia, cocoa powder, natural, and artificial flavors) (Juvenescence Ltd., Dublin, Ireland). We have previously reported on the kinetic profile (Crabtree et al., 2023) of this beverage containing a novel ketone diester (hexanoic acid and R‐butanediol), which after intestinal hydrolysis of BH‐BD, converts to R‐BHB and acetoacetate in the liver through the classical hepatic ketogenic pathway and alcohol dehydrogenase pathway, respectively. Two servings were consumed together (50 g total) to achieve the desired R‐BHB concentrations (Crabtree et al., 2023; Gormsen et al., 2017; Nielsen et al., 2019). A fat‐based placebo made in house was formulated with standard dietary ingredients (whey protein powder, cocoa powder, heavy cream, and canola oil) and matched to BH‐BD for volume and caloric content (Table 1).

**TABLE 1:** Nutritional information of both supplements.

	BH‐BD	PL
Volume (oz)	5	5
Ketone precursors (g)	50	0
Energy (kcal)	440	440
Protein (g)	4	4
Carbohydrate (g)	4	4
Fat (g)	0	44
Sodium (mg)	40	40

### 2.3 Participant preparation and baseline measures

Subjects reported to an outpatient imaging facility in the morning following a 12 h fast. Subjects were asked to abstain from alcohol for 24 h, caffeine for 10 h, exercise for 10 h, and to arrive adequately hydrated. A pregnancy test was administered to all female subjects of childbearing age.

At baseline, subjects completed an MRI screening form and a gastrointestinal (GI) symptom questionnaire; height and weight were measured for BMI calculation. Subjects then changed into MRI appropriate hospital gowns and an IV was placed in the antecubital vein for contrast administration and sequential blood draws. At baseline, blood was drawn for metabolic analysis and Glomerular Filtration Rate (GFR) testing. Following these tasks, the subject was taken into the MRI room, fitted with electrocardiogram (ECG) leads and an automated MRI‐conditional blood pressure (BP) cuff, and connected to an MRI compatible injection system (MEDRAD Spectris Solaris, Bayer Healthcare, Whippany, NJ). Baseline imaging scans were performed prior to ingestion of either beverage.

### 2.4 Beverage consumption and repeated measure data collection

After baseline imaging, the patient table was removed from the MRI bore, the subject sat up on the table and was given the study beverage (BH‐BD or placebo). Participants were required to fully consume the beverage within 15 min. A single 16 oz bottle of water was provided to each subject. Following beverage consumption, sequential blood draws, cardiac MRI (without contrast), BP measurements, and GI symptom assessments (scored 0–8: 0 = no symptom, 8 = unbearable symptom) were performed at the following timepoints post‐consumption: 15 min, 30 min, 60 min, and 90 min. At 120 min post consumption, a blood draw, GI symptom questionnaire, and cardiac MRI with contrast were performed. Immediately following the final scan, which took approximately 10 min, a final blood draw was taken.

### 2.5 Blood collection

Venous blood was collected sequentially throughout the study from an intravenous cannula placed in the antecubital vein. Approximately, 1 mL–2 mL of blood was drawn at each timepoint. The blood was immediately tested for R‐BHB and glucose content using a handheld glucometer (Keto Mojo, Keto‐Check Inc., Savannah, GA) and test strips.

### 2.6 Cardiac magnetic resonance imaging

All images were acquired using a 3T scanner (MAGNETOM Vida, Siemens Healthineers, Erlangen, Germany). At baseline and 120 min post‐consumption, a bolus injection of 0.075 mmol/kg of gadolinium‐based contrast agent (Gadavist, Bayer Healthcare, LC, Whippany, NJ) was administered using a power injector at a rate of 4 mL/s, followed by 20 mL of saline at 4 mL/s.

A full set of long axis and short axis cine images were acquired at each timepoint to measure LV and RV function (ventricular volumes, stroke volume, cardiac output, and ejection fraction). A prototype quantitative first‐pass perfusion sequence was used to measure global myocardial blood flow (MBF) and to estimate peak‐to‐peak pulmonary transit time (PTT) at baseline and 120 min post consumption (Kellman et al., 2017; Seraphim et al., 2021). Both LV and RV function were analyzed at each imaging timepoint using a commercial software (SuiteHeart, Neosoft LLC, Pewaukee, WI). MBF and PTT values were derived from the images acquired during the first‐pass of gadolinium following each bolus injection (Kellman et al., 2017). The arterial input function and dynamic myocardial enhancement were measured to generate the automated in‐line quantitative myocardial perfusion maps (Kellman et al., 2017). While MBF analysis was largely automated, manual adjustments were made to correct for motion‐induced slice misregistration. Pulmonary blood volume (PBV) was calculated as the product of PTT and CO (Seraphim et al., 2021). PTT and PBV underwent further analysis normalized to HR (PTTn and PBVn, respectively).

LV myocardial strain (peak and mean systolic longitudinal and circumferential) was estimated from short and long‐axis cine images using feature tracking software (SuiteHeart, Neosoft LLC, Pewaukee, WI). Peak strain represents maximal contractile function while mean strain provides a measure of overall deformation averaged throughout the cardiac cycle. Additionally, we calculated strain energy density using previously published equations (MacIver et al., 2022). Active Strain Energy Density (ASED) was calculated as an exploratory measure for longitudinal (GLASED), and circumferential (CASED) directions of myocardial force development at baseline and 120 min post‐consumption. ASED estimates the work done per unit volume of myocardium, combining contractile stress with percent shortening to provide a novel measure of myocardial mechanical work and contractile function that has shown to correlate with various cardiac disease states (MacIver et al., 2022).

### 2.7 Statistical analysis

Baseline demographics and characteristics for both groups were described using mean ± SD and were analyzed for statistical significance using independent sample *t*‐test. Venous blood ketone and glucose concentrations are reported as mean ± SD and were compared for time, supplement, and interaction (time*supplement) effects using a 2 × 6 repeated measures ANOVA. All raw measures of cardiovascular function were averaged across subjects in each group at each time point. These values were then analyzed as a percent change (calculated from the group averages) from baseline at each timepoint within each group. These were compared for time, supplement, and interaction (time*supplement) effects using a 2 × 6 repeated measures ANOVA. Myocardial perfusion ratio (MBF120min/MBFbaseline) is described using mean ± SD and compared between groups using independent sample *t*‐test. PTT, PTTn, PBV, and PBVn are compared between groups using independent sample *t*‐test of the normalized change score from 120 min post consumption to baseline. CASED and GLASED were compared between groups using normalized change scores from 120 min post consumption to baseline. These values were assessed for statistical significance using independent sample *t*‐tests. BP was compared between groups using a mixed effects 2 × 6 repeated measure ANOVA to account for missing data. The Mann–Whitney U test was performed to compare GI symptoms between conditions at each timepoint. Pearsons's correlation was used to assess the relationship between ketones and physiological variables. All statistics were performed in SPSS (ver. 29, IBM, NY). All variables of interest were screened for normality and homogeneity of variance using the Shapiro–Wilk test and Mauchly sphericity test. The dataset was assessed for any potential outliers by using the *z*‐score method using a *z* score threshold of 3; additionally, box plots were generated to visualize data sets to further identify outliers and confirm status. There were no outliers detected or removed from this dataset. Bonferroni correction was applied to all post hoc interpretations. Two‐tail alpha significance was set a priori at *p* ≤ 0.05.

## 3 RESULTS

### 3.1 Demographic and baseline characteristics

Twenty‐one subjects were recruited, enrolled, and successfully completed the study protocol. One subject was dropped from data analysis due to bouts of emesis during IV placement that occurred prior to ingesting the beverage at baseline, and again following BH‐BD ingestion. In the remaining 20 subjects (10 males, 10 females), there were no significant differences in baseline demographic and cardiac function measures between the BH‐BD and PL groups, but the PL group had slightly higher baseline venous blood glucose and R‐BHB concentrations (Table 2).

**TABLE 2:** Demographic and baseline characteristics.

	BH‐BD	PL	*p* Value
Demographics			
Sex (male/female)	5/5	5/5	n/a
Age (years)	34 ± 6	28 ± 8	0.06
Weight (Kg)	84 ± 8	78 ± 6	0.07
Height (cm)	176 ± 9	171 ± 7	0.16
BMI (kg/m^2^)	27 ± 2	27 ± 2	0.65
Metabolic measures			
BHB (mM)	0.3 ± 0.2	0.5 ± 0.2	0.04*
Glucose (mg/dL)	109 ± 14	124 ± 7	0.01**
LV function			
HR (bpm)	60 ± 10	64 ± 8	0.38
EF (%)	57 ± 4	55 ± 3	0.33
EDV (mL)	183 ± 29	196 ± 23	0.28
ESV (mL)	79 ± 17	88 ± 13	0.20
SV (mL)	104 ± 15	108 ± 12	0.53
CO (L/min)	6.2 ± 1.1	6.9 ± 1.1	0.16
RV function			
EF (%)	54 ± 5	53 ± 5	0.59
EDV (mL)	197 ± 38	212 ± 36	0.39
ESV (mL)	92 ± 25	102 ± 26	0.40
SV (mL)	105 ± 16	110 ± 13	0.46
CO (L/min)	6.2 ± 1.1	7.0 ± 1.1	0.11
LV myocardial strain			
Radial peak strain (%)	0.72 ± 0.1	0.70 ± 0.10	0.60
Radial mean strain (%)	0.27 ± 0.06	0.28 ± 0.04	0.70
Circumferential peak strain (%)	−0.16 ± 0.02	−0.15 ± 0.02	0.81
Circumferential mean strain (%)	−0.06 ± −0.02	−0.06 ± 0.01	0.59
Longitudinal peak strain (%)	−0.16 ± 0.02	−0.16 ± 0.02	0.98
Longitudinal mean strain (%)	−0.07 ± 0.02	−0.07 ± 0.01	0.75
Perfusion and flow			
MBF (mL/min*g)	60 ± 25	67 ± 29	0.58
PTT (s)	8.3 ± 2.3	7.5 ± 0.9	0.35
PBV	44 ± 9	45 ± 9	0.80
Blood pressure			
SBP (mmHg)	120 ± 9	120 ± 8	0.88
DBP (mmHg)	73 ± 10	66 ± 8	0.11
MAP (mmHg)	88 ± 9	84 ± 7	0.20

*Note*: Values reported as mean ± SD.

Abbreviations: BHB, beta‐hydroxybutyrate; BMI, body mass index; CO, cardiac output; DBP, diastolic blood pressure; EDV, end‐diastolic volume; EF, ejection fraction; ESV, end‐systolic volume; HR, heart rate; LV, left ventricle; MAP, mean arterial pressure; MBF, myocardial blood flow; PBV, pulmonary blood volume; PTT, pulmonary transit time; RV, right ventricle; SBP, systolic blood pressure; SV, stroke volume.

*p ≤ 0.05; **p ≤ 0.01.

### 3.2 Metabolic results

**FIGURE 2:**
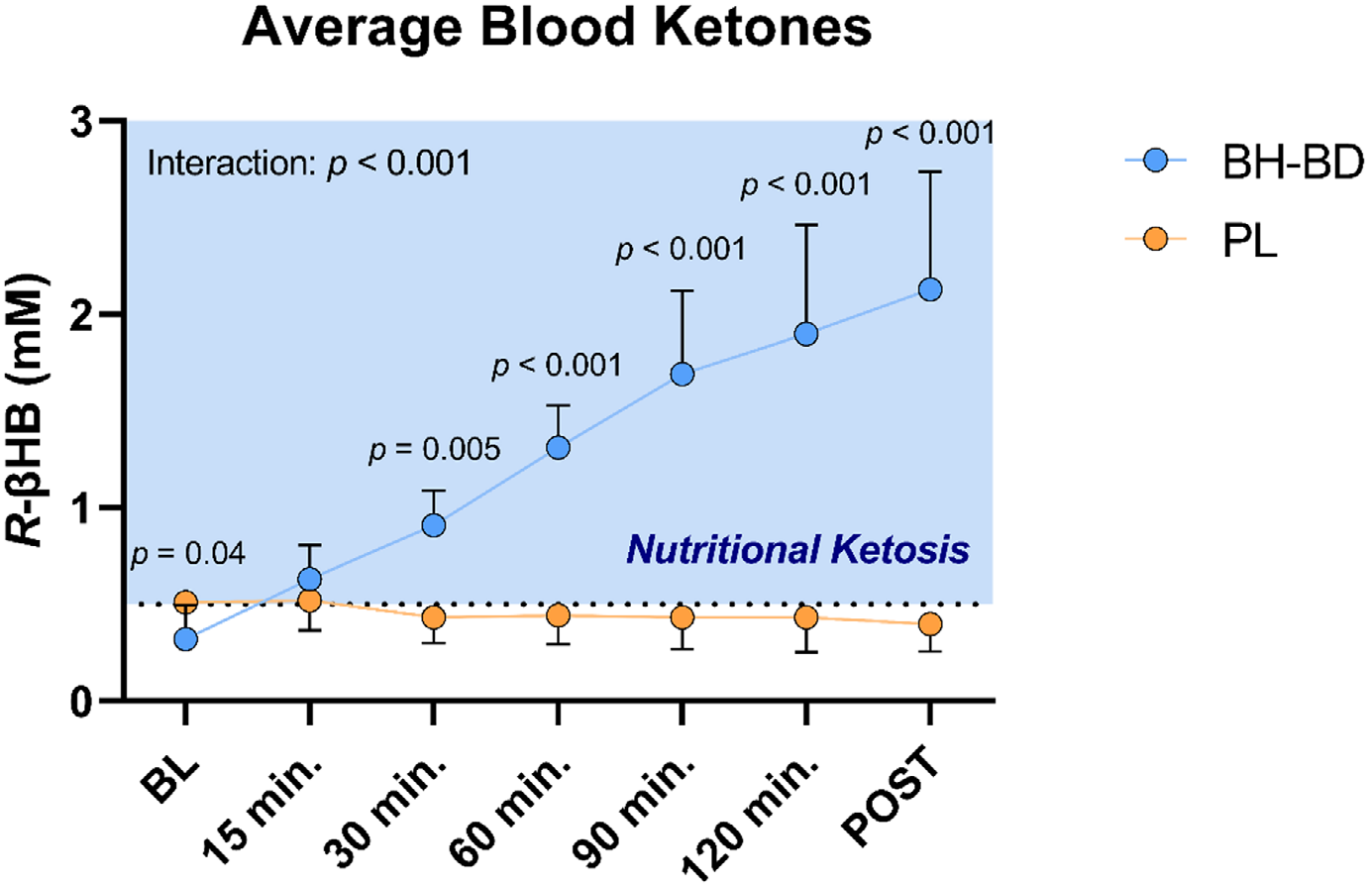
Venous blood *R‐*BHB responses to consumption of a ketogenic promoting beverage or a fat based, energy and volume matched, placebo. All values reported as mean ± SD. Between group *p* values are listed within timepoints with significant post‐hoc comparisons. BH‐BD, bis‐hexanoyl *R*‐1‐3‐butanediol; PL, placebo.

**FIGURE 3:**
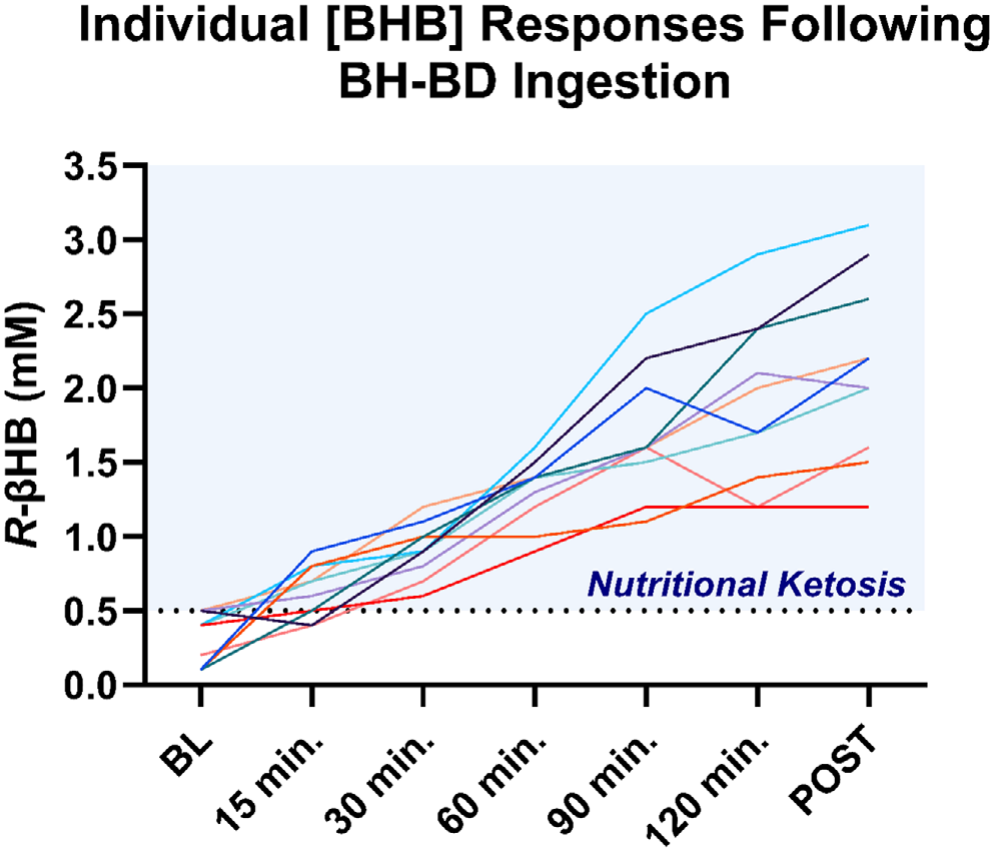
Individual venous blood *R‐*BHB response following BH‐BD consumption.

We were unable to obtain venous blood in one subject in the PL group. As expected, BH‐BD ingestion elicited a rapid elevation of [R‐BHB], compared to the PL group which showed a stable level of R‐BHB over the 2‐h postprandial period (Figure 2). Statistical analysis revealed significant main effects of time (*p* < 0.001) and supplement (*p* < 0.001), as well as a significant time*supplement interaction (*p* < 0.001) In the BH‐BD group, [R‐BHB] reached its highest value (2.1 ± 0.4 mM) following the final imaging timepoint. Although all subjects demonstrated a rise in blood [R‐BHB] after BH‐BD ingestion, there was a wide variation in peak [R‐BHB], ranging from 1.2 to 3.1 mM at the final time point (Figure 3). Final [R‐BHB] was not correlated with BMI (*r* = 0.48, *p* = 0.16) or body weight (*r* = 0.12, *p* = 0.72) in the BH‐BD cohort.

Only two of 19 subjects had glucose within the healthy fasting range (60–100 mg/dL) while the remaining subjects presented with a prediabetic glucose level (100–125 mg/dL). At baseline, glucose was lower in the BH‐BD group (Table 2). This difference persisted throughout the duration of the study (*p <* 0.001) leading to significant group (*p <* 0.001) and interaction (*p <* 0.001) effects. Glucose reduced following consumption of both beverages, favoring the BH‐BD (−18%) compared to the PL (−11%; *p <* 0.001).

### 3.3 Left ventricular function

**FIGURE 4:**
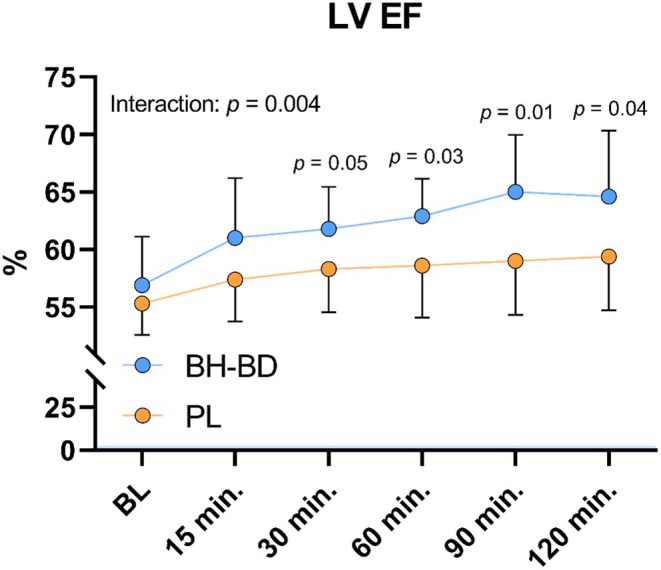
Left ventricle ejection fraction response following beverage consumption. All values reported as mean ± SD within group at each timepoint. Between group *p* values are listed within timepoints with significant post hoc comparisons. BH‐BD, bis‐hexanoyl R‐1‐3‐butanediol; EF, ejection fraction; PL, placebo; LV, left ventricle.

LV EF increased during the intervention (time effect: *p <* 0.001), particularly in the BH‐BD group (64.6 ± 5.7%) compared to PL (59.4 ± 4.7%) 2 h post‐consumption (group effect: *p* = 0.04), producing an interaction effect (*p* = 0.04) (Figure 4).

**FIGURE 5:**
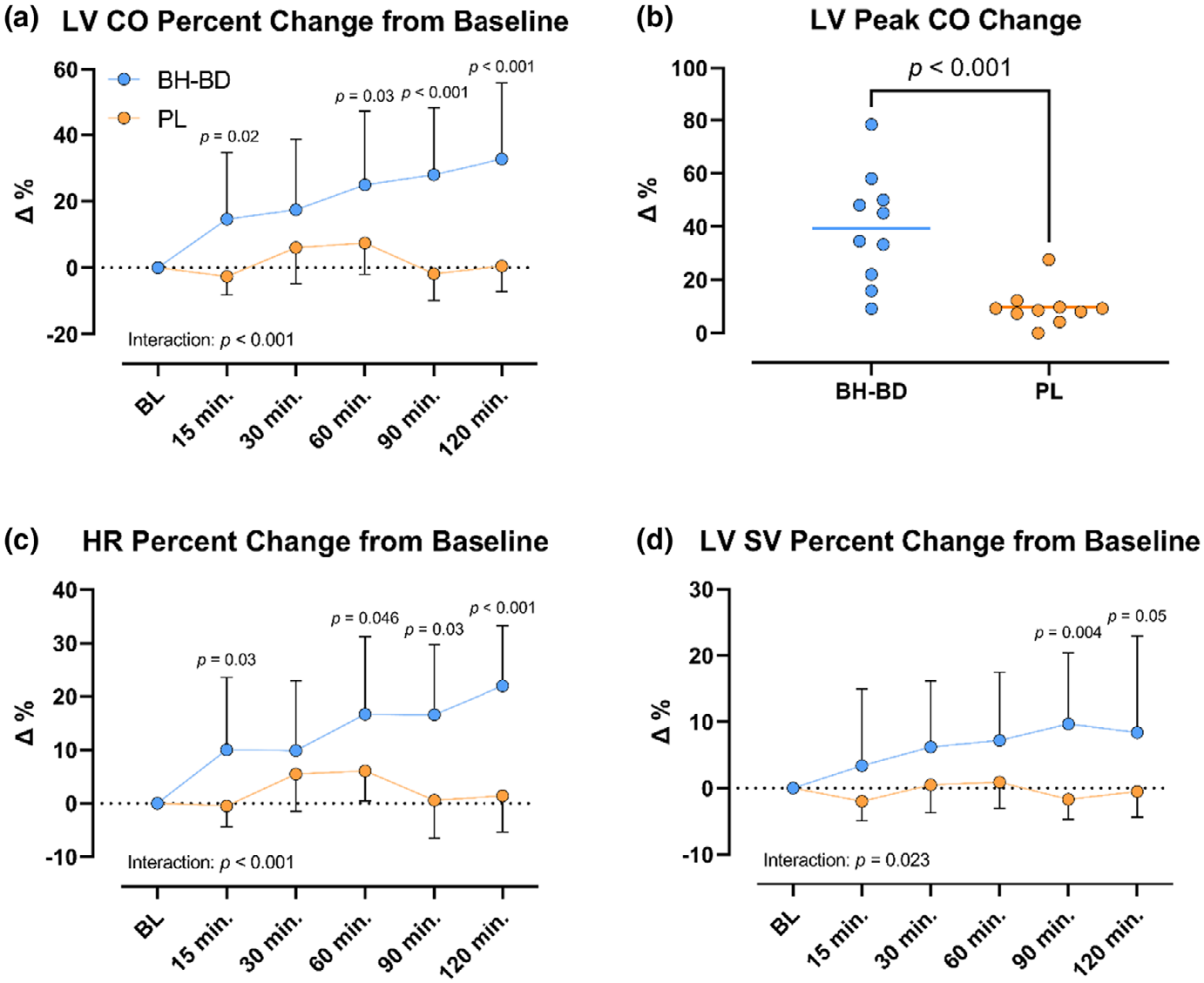
Left ventricular cardiac function measures at baseline and following beverage consumption. (a) LV cardiac output response as a percent change from baseline following BH‐BD and Placebo ingestion (b). LV peak cardiac output as a percent change from baseline represented individually within each group (c). Heart rate response as a percent change from baseline following beverage ingestion (d). LV stroke volume response as a percent change from baseline following beverage ingestion. All values reported as mean ± SD of the percent change from baseline within group at each timepoint. Between group *p* values are listed within timepoints with significant post hoc comparisons. BH‐BD, bis‐hexanoyl R‐1‐3‐butanediol; CO, cardiac output; PL, placebo; SV, stroke volume; LV, left ventricle.

Mean LV CO increased 31% 2 h following BH‐BD consumption while PL had no effect despite a transient nonsignificant response occurring at 30 min and 60 min, producing a significant interaction effect (*p <* 0.001) (Figure 5a). Mean peak change in LV CO was higher after BH‐BD than PL ingestion with considerable variation in the magnitude of change between subjects. Notably, all subjects in the BH‐BD group showed a peak change in LV CO greater than the mean value in the PL group (Figure 5b). Posthoc comparisons were significant at each timepoint following supplement consumption (*p <* 0.05) other than the 30 min timepoint. Elevation in LV CO was comprised of significant elevation in both HR (+22%, *p <* 0.001) and SV (11%; *p* = 0.028) (Figure 5c,d) in BH‐BD, while both were unchanged in PL (group effect: *p* = 0.04 and *p* = 0.004; respectively). HR (*p <* 0.001) and SV (*p <* 0.023) both produced significant interaction effects.

### 3.4 Right ventricular function

**FIGURE 6:**
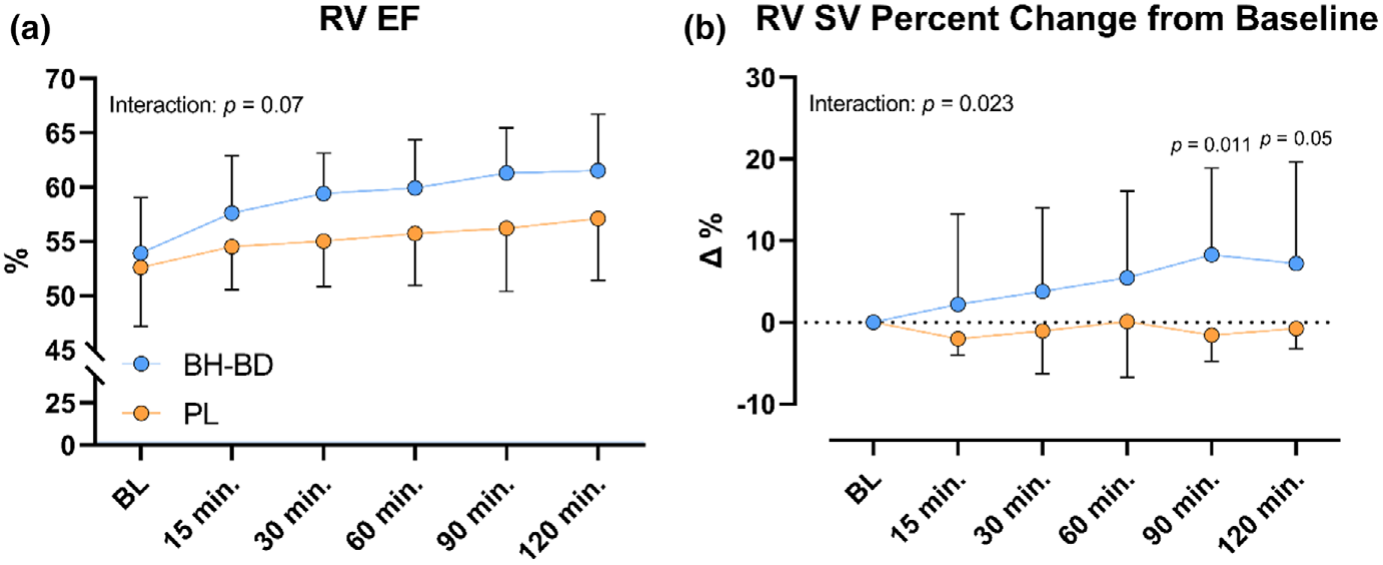
Right ventricular cardiac function measures at baseline and following beverage consumption. (a) RV ejection fraction (EF) response following supplement ingestion. (b) RV stroke volume response as a percent change from baseline following supplement ingestion. All EF values reported as mean ± SD within group at each timepoint. All SV values reported as mean ± SD of the percent change from baseline within group at each timepoint. Between group *p* values are listed within timepoints with significant post‐hoc comparisons. BH‐BD, bis‐hexanoyl *R*‐1‐3‐butanediol; EF, ejection fraction; PL, placebo; RV, right ventricle; SV, stroke volume.

RV EF significantly increased during the active intervention (*p <* 0.001) and trended toward significance between BH‐BD (120 min: 61.5 ± 5.2%) and PL groups (120 min: 57.1 ± 5.68%; *p* = 0.07). However, there were no pairwise differences between conditions at any timepoint (*ps* ≥0.08). RV CO had significant time (*p <* 0.001) and group (*p <* 0.001) effects following beverage consumption, resulting in a significant interaction effect (*p* = 0.003) as CO increased 30% with BH‐BD and PL was unchanged (Figure 6a). RV SV increased following beverage consumption (*p* = 0.02) in the BH‐BD group (*p* = 0.04) creating an interaction effect of time*supplement (*p* = 0.017) (Figure 6b).

### 3.5 LV myocardial strain

Peak and mean circumferential strain increased following BH‐BD consumption (*p <* 0.001) but there were no differences between groups, nor interaction effects. There was a significant increase of longitudinal strain, both peak (*p <* 0.001) and mean strain (*p <* 0.001), but only mean strain featured showed a significant interaction effect (*p* = 0.034). Further posthoc comparisons revealed that mean longitudinal strain was significantly different between groups at 90 min (*p* = 0.019) and 120 min (*p* = 0.042) following beverage ingestion.

### 3.6 ASED

There was a trend toward normalized GLASED improvement following BH‐BD (+20%) compared to PL (+6%) (*p* = 0.07). There was no difference between conditions in normalized CASED following BH‐BD (+21%) or PL (+8%) (*p* = 0.11).

### 3.7 Myocardial blood flow

**FIGURE 7:**
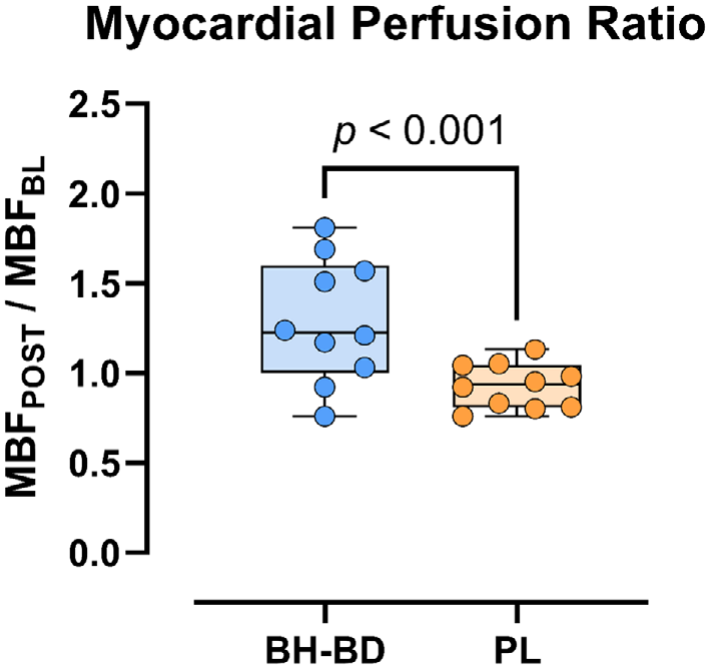
Myocardial perfusion ratio (baseline myocardial blood flow/post‐myocardial blood flow) following supplement consumption. BH‐BD consumption was associated with an elevation of myocardial blood flow compared to PL (*p* ≤ 0.001). Boxes represent the interquartile ranges. The middle line represents group median. Dots represent individual subject values.

Within‐subject myocardial perfusion ratio was significantly elevated following BH‐BD consumption (1.29 ± 0.34) compared to PL (0.93 ± 0.12) (*p* ≤ 0.001) (Figure 7).

### 3.8 Hemodynamics

No measures of cardiovascular hemodynamics were different at baseline between groups. PTT decreased 25% from baseline in BH‐BD (8.27 ± 2.32 s) to 2 h post consumption (6.20 ± 1.43 s) and was significantly different compared to PL which decreased 9% from baseline (7.51 ± 0.94 s) to 2 h post consumption (6.85 ± 1.14 s) (*p* = 0.007). Normalized to HR, PTTn decreased 39% from baseline 2 h following BH‐BD consumption compared to PL (*p* < 0.001).

There was a trend toward significant difference in pulmonary blood volume (PBV) change following supplementation between BH‐BD (+1%) and PL (−9%) (*p* = 0.07). PBV normalized to HR (PBVn) reduced (*p* = 0.002) but did not differ between groups (*p* = 0.26).

### 3.9 Blood pressure

There were no blood pressure (SBP, DBP, and MAP) differences between groups at baseline. SBP, DBP, and MAP remained stable during the intervention (time effects: *p* = 0.21; *p* = 0.61; *p* = 0.78, respectively) and did not differ between BH‐BD and PL groups (group effects: *p* = 0.70; *p* = 0.11; *p* = 0.16, respectively). As such, SBP, DBP, and MAP did not feature interaction effects (*p =* 0.98; *p* = 0.51; *p* = 0.84, respectively).

### 3.10 GI symptoms

Subjects experienced some mild nausea at 60 min following BH‐BD ingestion (median 0; range 1–2; *p* = 0.03). There was also a trend for mild nausea at 90 min (median 0; range 1–2; *p* = 0.068) and 120 min (median 0; range 1–2; *p* = 0.068) and mild bloating at 90 min (median 0; range 1–2; *p* = 0.068; *p* = 0.067) following BH‐BD ingestion. There were no symptoms classified greater than “mild” other than “urge to urinate” which occurred following both beverages (*p* > 0.05).

## 4 DISCUSSION

The primary aim of this pilot study was to examine the acute impact of BH‐BD consumption on cardiovascular function in adults with no history of cardiovascular disease. To capture the comprehensive cardiopulmonary response to exogenous ketones, we conducted simultaneous evaluations of biventricular cardiac function, myocardial perfusion, and pulmonary transit time with cardiac MRI. As a comparison, this study implemented a fat‐based placebo, energy and volume matched to the ketogenic‐promoting beverage, to control for the effects of fluid and energy intake on cardiovascular function (Hauser et al., 2016). We implemented an advanced multi‐parametric cardiac MRI protocol over the course of 2 h, doubling the duration of the previously reported longest acute imaging study (Oneglia et al., 2023) to comprehensively investigate the dynamic cardiovascular response to ketone availability. This extended timeline with repeated imaging allowed us to track the trajectories of cardiac changes, enabling the identification of change in individual aspects of the cardiovascular response, while also revealing temporal relationships between various functional parameters.

Additionally, we investigated a ketone diester supplement, rather than ketone monoester (Oneglia et al., 2023; Selvaraj et al., 2022). Ketone diesters in comparison to ketone monoesters are metabolized more slowly providing a longer sustained ketone elevation which may be more beneficial in free‐living contexts, providing longer daily ketone exposure and organ uptake (Evans et al., 2022). Ingestion of BH‐BD elicited a rapid R‐BHB elevation within 15 min, without reaching a discernable kinetic peak 120 min post‐ingestion. There was a large intersubject variation in ketone kinetics that was not due to physiological variables assessed here (BMI and body weight) despite administration of a standardized BH‐BD serving. Compared to a placebo matched for energy and fluid volume, BH‐BD ingestion significantly increased cardiac output, myocardial blood flow, and LV systolic strain over the 2‐h postprandial period. These results highlight the potential of exogenous ketones and ketone promoting beverages to acutely augment resting cardiovascular function and myocardial blood flow for a period of hours in individuals with normal heart function.

Cardiac output acutely elevated within 15 min following BH‐BD consumption, when blood R‐BHB had only slightly exceeded 0.5 mM, and then continued increasing in a dose–response manner through the 120 min imaging timepoint along with progressively higher BHB concentrations. Following BH‐BD ingestion, the highest [R‐BHB] and CO were measured at the final 120 min timepoint leaving open the possibility that we did not capture the true peak in either parameter. The magnitude of CO increase (~30%) seen in this largely prediabetic cohort was similar to previous exogenous ketosis trials (~20%–40%) in studies of ingested BH‐BD in healthy adults (Selvaraj et al., 2022), as well as ketone salt infusion in people with HF and age‐matched controls (Nielsen et al., 2019). SV and HR elevation at the highest [R‐BHB] was likewise consistent with previous examinations of cardiovascular response to acute exogenous ketosis (Gormsen et al., 2017; Nielsen et al., 2019; Selvaraj et al., 2022). Of note, CO elevation was attributed to both HR and SV elevation, in a manner resembling cardiovascular response to exercise, whereby both HR and SV rapidly increase to boost cardiac output until SV plateaus (Astrand et al., 1964). One exogenous ketosis study performed in people with acute cardiogenic shock alongside standard of care emergency drug administration did not find an increase in HR (Berg‐Hansen et al., 2023), while others have found reduced contribution from SV in young healthy adults (Oneglia et al., 2023), despite both studies finding elevated CO. This discrepancy likely results from frequent tachycardia experienced with cardiogenic shock, alongside other administered drugs, elevating HR prior to ketone administration (Bertini & Guarracino, 2021). LVEF has been reported to increase in healthy adults (Selvaraj et al., 2022) and people with HF (Nielsen et al., 2019) following supplementation, similar to our findings, in addition to our novel observation of RVEF elevation, suggesting ketones impact overall cardiopulmonary function rather than selectively targeting LV function and its primary determinants, such as systemic vascular resistance (SVR) or LV coronary perfusion. Given the interest in ketone therapy for a wide variety of cardiovascular diseases, it is crucial to understand the cardiac response across the entire heart in a biventricular manner, not just the LV.

Interestingly, the PL elicited a marginal transient increase of CO at the 30 min and 60 min timepoints comprised primarily of HR elevation. The elevation and timeline are in line with previous postprandial results that utilized a calorie (1635 kcal) and macronutrient dense beverage food‐stress protocol (Hauser et al., 2016). Given that caloric load and intake volume were matched between supplements in this study, we provide evidence that the observed cardiac effects were attributed to an elevation in R‐BHB rather than simple fluid or caloric intake.

While we and others found no change in MAP (Nielsen et al., 2019), a decrease in SVR has been previously reported (Nielsen et al., 2019; Selvaraj et al., 2022). The reduction in PTT, while not a direct measure, suggests an acute elevation in preload, diastolic function, and overall lung circulation (Seraphim et al., 2021). This hemodynamic response persisted when normalized to heart rate, controlling for the physiological chronotropic effect of exogenous ketones. In addition to our findings with PTT, others have observed improvements in pulmonary pressure and function following 2 weeks of repeated daily exogenous ketone consumption in people with HF, manifesting during both rest and exercise (Berg‐Hansen et al., 2024).

Myocardial strain is gaining importance for early diagnosis, prognosis, and as a guide for therapeutic decision‐making (Rajiah et al., 2022) due to its sensitivity to subtle changes in cardiac function prior to observable changes in EF% (Brady et al., 2023). Both longitudinal and circumferential myocardial strain increased during the intervention, but only longitudinal myocardial deformation differed between supplement groups. Interestingly, longitudinal deformation began to differentiate between groups during the SV plateau phase of the CO elevation response to increased [R‐BHB], in a manner similar to exercise. At this point, myocardial deformation began to differ between groups, suggesting the cardiomyocytes were generating more force than previously, due to either (1) the afterload of the vasculature, (2) a change in inherent contractility, or (3) an increase in preload via the Frank‐Starling mechanism (Chitiboi & Axel, 2017). SVR as noted previously, decreases during exogenous ketosis (Nielsen et al., 2019; Selvaraj et al., 2022), but has not been examined in this context during the SV plateau phase of CO elevation in presence of increased [R‐BHB]. It is apparent that the initial increase in strain can be attributed, at least in part, to increased preload, as detailed here, but does not explain the differences during the SV plateau phase. There is some evidence that contractility increases with exogenous ketosis (Selvaraj et al., 2022), including in COVID‐19 (Wodschow et al., 2023) and people with HF (Nielsen et al., 2019), but detailed investigations are scant. Here, we corroborate these findings with novel exploratory evidence of a trend toward increased ASED, a measure of work standardized to unit of myocardium, suggesting possible improved contractility, in addition to improved EF of both left and right ventricles. We demonstrate a potential inotropic effect of ketones augmenting cardiomyocyte contractility, beyond just vasodilation, that requires further investigation.

Global LV MBF increased markedly following BH‐BD consumption, similar in scale to the elevation in CO. There was a wide degree of MBF variability at baseline between subjects, as has been reported previously in healthy adults (Chareonthaitawee, 2001). Our rest perfusion results generally align with the BHB infusion trial conducted by Nielsen et al. (2019), but are approximately half the effect seen by Gormsen et al. (2017); it is not immediately clear the source of these discrepancies. Methodologically, both infusion trials quantified myocardial perfusion with PET‐CT, while we used a novel cardiac MRI sequence (Kellman et al., 2017), but these techniques have previously shown good agreement in various physiological states (Engblom et al., 2010). Given that cardiac function results are remarkably consistent between ketone delivery methods (beverage vs. infusion), specific ketone chemical compound, population, and imaging modality, these discrepancies in MBF between studies are noteworthy and require further investigation. Notably these changes occurred in the context of physiological rest in the supine position and are reduced in comparison to perfusion responses to exercise or pharmacologic vasodilation.

Subjects consumed a 50 g BH‐BD serving size which resulted in blood [R‐BHB] levels close to those reported previously to increase cardiac output (Gormsen et al., 2017), while maintaining tolerability (Crabtree et al., 2023). While we observed lower peak [R‐BHB] on average compared to previous infusion and supplement trials, we observed similar changes in cardiac function (Gormsen et al., 2017; Nielsen et al., 2019; Selvaraj et al., 2022) along with a potential dose response relationship (Nielsen et al., 2019) reflecting CO and [R‐BHB] both steadily increasing over the 2 h intervention. It should also be noted that nine of 10 BH‐BD subjects recorded the highest [R‐BHB] at the final post‐cardiac MRI timepoint, so it remains unknown whether there was a continued increase after this timepoint.

Mild symptoms were reported by only two subjects in the BH‐BD group; all others were symptom‐free. There was a wide degree of variability in [R‐BHB] response across the subjects, which previous investigations have not been able to explain fully by physiological factors (body weight, BMI, sex, or age) nor diet (Crabtree et al., 2023; Shivva et al., 2016). More research is needed to better understand the physiological mechanisms that account for differential R‐BHB response to acute exogenous ketosis, especially considering the growing interest in clinical use of these beverages.

While the cardiovascular response to exogenous ketosis is becoming increasingly well characterized in the resting fasted state in a laboratory, no evidence exists regarding the response if consumed in a free‐living context, such as during submaximal exercise (i.e., free‐living, walking, and using stairs) when cardiovascular performance is elevated, or during the postprandial state. These questions are foundational to our clinical understanding of these potential therapeutic agents. Individuals with heart failure have significantly reduced cardiac reserve (Borlaug et al., 2010), and elevated resting function may be undesirable without a concomitant increase in maximal function. While BHB kinetics are suppressed when exogenous ketones are consumed with a meal (Stubbs et al., 2017) or unchanged compared to the fasted state (Crabtree et al., 2023), nutritional ketosis is still achieved, and this effect may augment depending on the macronutrient makeup of the meal; no studies thus far have examined the cardiovascular response to exogenous ketones when consumed with a meal, which would provide translational value for free living contexts, presumably the situation these agents would be employed. One study has examined the effect of daily, free‐living, exogenous ketone consumption in people with HF and found persistent cardiopulmonary improvements to CO, filling pressures, and cardiac volumes, following 2 weeks (Berg‐Hansen et al., 2024). There is a need for cardiovascular characterization in a variety of physiological states following ketone consumption or promotion before true translational impact can be determined.

Collectively, these results highlight the impactful and pleiotropic effects of acute exogenous ketosis on cardiovascular and pulmonary function. Thus far, limited evidence has suggested these acute effects confer clinical value during acute health episodes (Berg‐Hansen et al., 2023) that extend to chronic disease improvement following daily consumption for short timeframes (Berg‐Hansen et al., 2024). Further investigation must be completed to assess patient outcomes over longer supplementation periods to examine clinical efficacy and impact.

There were a few limitations in this study. Enrolled subjects spanned a wide range of fasting glucose levels. While it may have been advantageous to enroll only subjects with normal fasting glucose to reduce variability in metabolic health status and thus potential response to ketone availability, the cardiovascular responses observed here in a largely prediabetic cohort are in line with similar studies conducted in patients and healthy adults. The study timeline and product serving size were intended to reach similar R‐BHB levels as previously reported, but we did not capture R‐BHB peak within 2 h, and therefore likely also did not capture peak cardiovascular response. We did not measure central venous pressure, thus were unable to calculate SVR, which would have provided a more definitive explanation for the mechanism responsible for observed elevation in myocardial strain and work. A crossover design where each subject served as their own control would have been preferred, but we were able to enroll a relatively well‐matched control group to minimize the chances of any differences in subject characteristics between groups driving differential cardiovascular responses to BH‐BD versus PL in addition to conducting within‐subject analysis with serial cardiac MRI.

In conclusion, acute BH‐BD consumption gradually increases circulating blood R‐BHB over 2‐h concomitant with stepwise elevations of multiple indices of cardiac function, myocardial blood flow, and LV systolic strain in a group of individuals without cardiovascular disease.

## AUTHOR CONTRIBUTIONS

C.D.C., J.V., and O.P.S. conceived and designed research. C.D.C., Y.P., T.B., and O.P.S. performed experiments. C.D.C., P.C., Y.W., and Y.H. analyzed data. C.D.C., Y.H., J.V., and O.P.S. interpreted results of experiments. C.D.C., J.S., and A.B. prepared figures. C.D.C. drafted manuscript. C.D.C., Y.P., P.C., Y.W., D.S., T.B., J.S., D.D., M.K., B.R., A.B., Y.H., J.V., and O.P.S. edited and revised manuscript. C.D.C., Y.P., P.C., Y.W., D.S., T.B., J.S., D.D., M.K., B.R., A.B., Y.H., J.V., and O.P.S. approved final version of manuscript.

## FUNDING INFORMATION

The study was supported in part by a Department of Defense grant W81XWH‐22‐1‐0867 awarded to the Ohio State University (Y.H., J.V., and O.P.S.). Study product and placebo were provided by Juvenescence.

## CONFLICT OF INTEREST STATEMENT

Dr. Simonetti receives funding from Siemens through an institutional grant.

## ETHICS STATEMENT

The study was conducted according to the guidelines of the Declaration of Helsinki, and approved by the Institutional Review Board (or Ethics Committee) of The Ohio State University (2021H0425; 4/01/2022). Informed consent was obtained from all subjects involved in the study.

## Data Availability

Data can be made available upon request to the corresponding author.
